# Prognostic Role of OX40, LAG-3, TIM-3 and PD-L1 Expression in Bone and Soft Tissue Sarcomas

**DOI:** 10.3390/jcm13123620

**Published:** 2024-06-20

**Authors:** Bediz Kurt İnci, Elif Acar, Fatih Gürler, Ayşegül İlhan, Fatih Yıldız, Fisun Ardıç, Berna Öksüzoğlu, Nuriye Özdemir, Ahmet Özet, Güldal Esendağlı, Ozan Yazıcı

**Affiliations:** 1 Medical Oncology Department, Gazi University Hospital, 2906500 Ankara, Turkey; fatih_gurler@yahoo.com (F.G.); nyozdemir@yahoo.com (N.Ö.); ahmetozet@gmail.com (A.Ö.); drozanyazici@gmail.com (O.Y.); 2 Pathology Department, Gazi University Hospital, 2906500 Ankara, Turkey; e.elifacar@hotmail.com (E.A.); drguldal@yahoo.com (G.E.); 3 Medical Oncology Department, Dr. Abdurrahman Yurtaslan Ankara Oncology Hospital, 2906200 Ankara, Turkey; ayse_ilhan85@hotmail.com (A.İ.); dr.fatihyildiz@hotmail.com (F.Y.); bernaoksuzoglu@yahoo.com (B.Ö.); 4 Pathology Department, Dr. Abdurrahman Yurtaslan Ankara Oncology Hospital, 2906200 Ankara, Turkey; fisun01@gmail.com

**Keywords:** sarcoma, OX40, TİM-3, LAG-3, PD-L1

## Abstract

**Introduction:** The current study aims to evaluate the OX40, TIM-3, LAG-3, and PD-L1 targeted pathways in the regulation of T-cell activity in sarcoma patients to determine their relationship with overall survival (OS). **Method:** This study included one hundred and eleven patients with bone and soft tissue sarcoma diagnosed in two centers between 2010 and 2020. OX40, LAG-3, TIM-3 and PD-L1 expression levels were evaluated immunohistochemically from pathology preparations. **Results:** PD-L1 staining was detected in tumor cells, OX40, LAG-3, TIM-3 staining was detected in inflammatory cells in tumor tissue. In univariate analysis, no significant relationship was found between OX40, TIM-3, LAG-3, and PD-L1 staining and overall survival (respectively: *p* = 0.12, *p* = 0.49, *p* = 0.31, *p* = 0.95). When grade and stage at diagnosis, which were found to be significant in univariate analysis, along with OX-40, TIM-3, LAG-3, and PD-L1, were evaluated in multivariate analysis, a positive effect of OX-40 staining on overall survival was determined (*p* = 0.009). Considering the correlation between PDL-1 and OX40, TIM-3, and LAG-3 staining, a significant positive correlation was found between PDL-1 and TIM-3 and LAG-3 staining (respectively; *p* = 0.002, *p* = 0.001). **Conclusions:** There was no significant relationship between the PDL-1 staining percentage of tumor cells and OX40, TIM-3, and LAG-3 staining in inflammatory cells with the OS of sarcoma patients. However, detecting a significant positive correlation between PDL-1 staining and TIM-3 and LAG-3 staining also holds promise for finding effective targetable combination therapies that can prolong survival in sarcoma patients in the future.

## 1. Introduction

Cancer immunotherapy has changed the treatment landscape in oncology, modified the therapeutic algorithms for multiple malignancies, and become the leading treatment for metastatic diseases [1]. This success could not be achieved in bone and soft tissue sarcomas (STS) except for some subtypes [2].

It is a known fact that the inhibition of T-cell activation and proliferation and the escape mechanisms of tumor cells from the immune system effectively decrease conventional treatment responses in cancer patients [3]. Therefore, understanding the mechanisms of immune escape is crucial not only for enhancing the effectiveness of existing cancer treatments but also for developing new therapeutic approaches in immunotherapy. These mechanisms, co-inhibitors of T-cell activity, programmed cell death receptor-1 (PD-1), and cytotoxic T-lymphocyte-associated antigen 4 (CTLA-4), are the first targetable immune pathways detected in the era of immunotherapy. Immunotherapies are still predominantly carried out through PD-1, PD-1ligand (PD-L1), and CTLA-4. PD-1 is a cell-surface protein receptor expressed in B lymphocytes, activated T lymphocytes (CD4-8), and NK cells. Although PD-L1 is expressed in epithelial and endothelial cells, it can be expressed in tumor cells, especially in order to evade immune response [4].

In 2015, the application of 50% PD-1/PD-L1-positive lymphocytes in the bone and STS tumor microenvironment (TME) was carried out by Movva et al. In 2018, in a meta-analysis of 14 articles containing 15 independent studies and 1451 patients, PD-L1 expression level was associated with poor prognosis and poor disease-free survival (PFS) in STS [5]. With these results, the PD-1 inhibitor pembrolizumab was tested in bone and STS in the SARC028 study in 2017. Although pembrolizumab showed efficacy in some subtypes, undifferentiated pleomorphic sarcoma (UPS) (RR 40%) and liposarcoma (RR 20%), it could not show efficacy in most subtypes (synovial sarcoma, leiomyosarcoma, and bone sarcoma) and did not meet the endpoint overall response rate (ORR) [2]. Combination therapies have been started to be tried with the limited efficacy of immunotherapy monotherapies. The combination of pembrolizumab with cyclophosphamide did not provide significant efficacy in ORR and overall survival (OS), but the combination with first-line doxorubicin is promising [6,7]. In another combination study, drugs called nivolumab, which binds to PD-1 and blocks ligand interaction, and ipilimumab, which binds to CTLA-4 increased ORR, have been used [8].

The lack of desired success in sarcoma patients in the era of immunotherapy has led sarcoma studies to identify new targets. In other malignancies like breast cancer and lung cancer, co-stimulator OX40 and co-inhibitors T-cell immunoglobulin, mucin domain-3 (TIM-3), and lymphocyte activation gene-3 (LAG-3) are also involved in immune response regulation [9,10]. Therefore, it is important to investigate the relationship between different immunotherapy pathways in sarcoma and bone cancer patients to achieve satisfactory immunotherapy responses. The main rationale for this is that immunotherapy combination treatments have increasingly gained importance in preventing immune escape in recent years.

OX-40R (CD134) is a membrane-associated glycoprotein in the TNF receptor family. The OX-40R/OX-40L interaction enhances the survival of regulatory T-cells (Treg) and develops a suppressive response against anti-tumor immunity [11]. To the best of our knowledge, only one study in the literature has evaluated OX40 immunohistochemically and investigated its relationship with prognosis in sarcoma patients [12]. The study had a small number of patients and a limited sarcoma subtype, although OX40 staining was detected. As a result, its relationship with prognosis could not be determined. Other studies in the literature on OX40 and sarcoma are preclinical [13,14].

LAG-3 protein binds to major histocompatibility complex 2 (MHC class II), inhibiting the secretion of interferon-gamma (IFNγ) from antigen-specific effector T cells and attenuating anti-tumor immunity [15]. TIM-3 is expressed on CD4+ and CD8+ T lymphocytes and is associated with immunosuppression in the TME and poor prognosis in cancer patients [16]. The most comprehensive study on LAG-3 and TIM-3 in sarcoma patients showed their staining in various sarcoma subtypes, and their co-expression with PD/PD-L1 was determined. Although the total number of patients was high in the study, sarcoma subtypes included a limited number of patients, and no information was given about their relationship with prognosis [17].

Drugs targeting these pathways are in active development in multiple cancer subtypes [18], and anti-TIM-3 antibodies have shown some promise in murine sarcoma models in preclinical trials [19]. Likewise, the efficacy of the LAG-3 inhibitor has been demonstrated in a preclinical sarcoma trial, and clinical trials are still ongoing [20].

A limited number of studies have examined OX40, LAG-3, and TIM-3 immune pathways in sarcoma [12,13,17]. As a result of these studies, it has been determined that OX40, TIM-3, and LAG-3 expression are particularly present in some subtypes of sarcoma. However, there are not yet enough studies available to clearly define the role of these targets in clinical outcome. Identifying the prognostic role of OX40, TIM-3, and LAG-3 thoroughly will pave the way for testing new treatment modalities targeting them.

Response rates and survival in the treatment of soft tissue and bone sarcomas were low until the mid-2010s. Although PD-1/PD-L1- and CTLA-4-targeted cancer immunotherapy has achieved impressive results in many types of cancer, the desired success has not yet been achieved in sarcoma. Since sarcomas are immune cold tumors and immune escape mechanisms are thought to play a role in this failure, studies in this area have begun in the treatment of sarcoma [21]. This situation has revealed the need to find new targetable pathways and the necessity of combination treatments. In our study, we first aimed to examine the presence of OX40, TIM-3, LAG-3, and PD-1/PD-L1 targets in sarcoma patients and their relationship with survival. In recent years, combination immunotherapy treatment approaches have gained attention for increasing treatment responses. Therefore, as a secondary aim in our study, we aimed to evaluate the positive correlation between OX40, LAG-3, and TIM-3 with PD-L1 expressions. The correlation of OX40, LAG-3, and TIM-3 with PD-L1 targets in the development of combination treatments that could enhance treatment responses in sarcoma patients shows promise.

## 2. Materials and Methods

### 2.1. Patient Selection

The ethics committee approval of our study was granted by the Clinical Research Ethics Committee of Gazi University Hospital on 3 October 2018 and numbered 776. The clinical data and pathology materials of 111 patients with soft tissue and bone sarcomas treated at Gazi University Hospital and Dr. Abdurrahman Yurtaslan Ankara Oncology Hospital between 2010 and 2020 were retrospectively reviewed. Osteosarcoma and chondrosarcoma subgroups were evaluated as bone sarcomas. Patients with sarcoma types Ewing’s sarcoma and gastrointestinal stromal tumors whose clinical course and treatment schemes are fundamentally different from other sarcomas were excluded.

### 2.2. Pathological Evaluation

Tissue blocks from the pathology archives of two centers were used in the study. The cases were reviewed at the beginning of the study to confirm the diagnosis. Surgically resected tissue blocks (nonmetastatic disease) and biopsies of the metastatic region (metastatic disease) were used. Immunohistochemistry staining of formalin-fixed paraffin embedded (FFPE) tumor tissues (4 mm whole sections) was performed using a Ventana Benchmark XT automated staining platform (Ventana Medical Systems) with the antibodies. Stained slides were reviewed and scored by a single pathologist. Inflammatory cells within the tumor, tumor cells, and in cases where non-tumor tissue was present, inflammatory cells in the surrounding non-tumor tissue were evaluated for each case.

#### 2.2.1. PD-L1 Assessment

A PD-L1 IHK/ISH test was stained with OptiView DAB IHK Detection Kit on VENTANA PD-L1 (SP263) Assay (Provider: Roche Diagnostics), and PDL-1 was evaluated qualitatively. While evaluating PD-L1 staining in tumor cells, all sections were scanned with a 20× objective (×200 magnification). The ratio of tumor cells with membranous staining to all tumor cells is expressed as a percentage and scored on a continuous basis from 0 to 100% and considered positive in the context of ≥1% staining. These percentages were divided into 2 groups: <1% and ≥1%. In PD-L1-stained tumors, the staining intensity was heterogeneous and was detected in varying intensities, from mild to strong. While evaluating PD-L1 staining in inflammatory cells within the tumor, all sections were scanned with a 20× objective (×200 magnification). Cytoplasmic granular staining and membranous staining were considered positive. The ratio of stained inflammatory cells to all inflammatory cells was evaluated as a percentage. A percentage of 1% and above was considered positive.

#### 2.2.2. TIM-3 and LAG-3 Evaluation

TIM-3 was stained with rabbit monoclonal antibody (D5DSR), and LAG-3 was stained with recombinant anti-LAG-3 antibody [EPR20261] (ab209236)-Abcam rabbit monoclonal antibody with the OptiView DAB IHK Detection Kit on a Ventana closed-system device.

TIM-3 and LAG-3 staining was evaluated in inflammatory cells within the tumor, and stained inflammatory cells were counted in a high magnification area (40° objective = 400 magnification) with the highest staining. Membranous, cytoplasmic, and Golgi zone staining was considered positive.

TIM-3 staining in tumor cells was evaluated as present/absent (+/−). It was observed that there was generally focal and weak intensity staining in the cells of the tumor stained with TIM-3. LAG-3 staining was not observed in tumor cells. Values given for TIM-3 and LAG-3 were calculated using the average of 3 high power fields, and ≥1 inflammatory cell was considered positive.

#### 2.2.3. OX40 Assessment

OX40L receptor antibody (ab203220) was stained with Abcam and OptiView DAB IHK Detection Kit on Ventana closed-system device.

When evaluating OX40 staining in inflammatory cells within the tumor, stained inflammatory cells were counted in a high magnification field containing the most staining (40 objective = 400 magnification). Membraneous staining was considered positive. The entire section was scanned, and the values given for OX40 were calculated using the average of 3 high power fields.

### 2.3. Statistical Analysis

Statistical analyses were performed using SPSS version 21.0, and *p* < 0.05 was considered statistically significant. Bivariate correlation analysis and Kaplan–Meier survival analysis was used to determine the relationship between pathological staining and survival. Chi-Square tests were used to determine the relationship between two verbal variables. A independent-sample T-test was used to determine the relationship between verbal and numerical variables.

## 3. Results

Pathology samples and clinical data of 111 soft tissue and bone sarcoma patients diagnosed between 2010 and 2020 were screened retrospectively. The patients’ mean age (±std) was found to be 45.4 ± 1.7. Other characteristic features of the patients are presented in Table 1. Subtypes and frequencies of soft tissue and bone sarcomas are given in Table 2.

In the survival analysis of the patients, the median OS was found to be 35 months (IQR 15–63) (min-max 1–230) in the whole group, and the median OS was 10 months (IQR 4–17) (min-max 1–128) in the metastatic disease group (Table 1). Of 100 patients in the local stage at diagnosis, 46 had metastatic recurrence.

PDL-1 is expressed in tumor cells, whereas significant staining could not be detected in tumor cells with OX40, TIM-3, LAG-3. OX40, TIM-3, and LAG-3 stainings were observed and evaluated in tumor-infiltrating lymphocytes. All sarcoma staining with OX40 was 18%, with TIM-3 was 36%, and with LAG-3 was 36.9%. The staining percentages according to sarcoma subtypes are detailed in Table 3.

When evaluating factors affecting overall survival, univariate analyses revealed that the metastatic stage at diagnosis and histological high grade were found to have a negative impact on overall survival. In multivariate analysis, in addition to the stage at diagnosis and histological grade, it was also determined that OX40 staining in inflammatory cells within the tumor tissue effected survival (Table 4).

**Figure 1:**
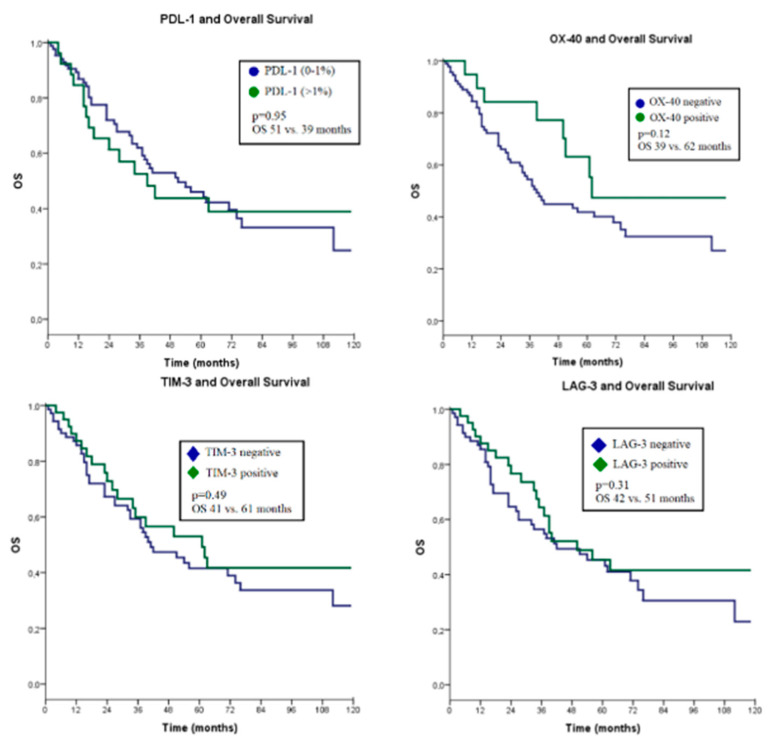
Overall survival analysis.

The Kaplan–Meier survival analysis revealed no significant correlation between the percentage of PDL-1 in tumor cells and the staining of OX40, TIM-3, and LAG-3 in inflammatory cells within the tumor tissue with overall survival (*p* = 0.95, *p* = 0.12, *p* = 0.49, *p* = 0.31, respectively) (Figure 1). A correlation between PDL-1 and OX40, TIM-3, LAG-3 stainings was examined using the Chi-square test. Significant positive correlations were found between PDL-1 staining and TIM-3 as well as LAG-3 staining (respectively: *p* = 0.002, *p* < 0.001) (Table 5).

## 4. Discussion

In the era of immunotherapy, the role of targetable immune pathways in many types of cancer has been clearly observed in treatment. However, treatment responses are still not at the desired level in soft tissue and bone sarcomas, especially in advanced-stage disease. The current study demonstrated that OX40 staining in inflammatory cells within the tumor tissue effected survival, and positive correlations were found between PDL-1 staining and TIM-3 and LAG-3 staining.

Our study, similar to the literature regarding histological subtypes, predominantly includes leiomyosarcoma in soft tissue sarcomas and osteosarcoma in bone sarcomas [22]. Despite recent advancements in treatment, the overall survival for unresectable sarcomas still ranges between 15 and 20 months [23,24,25]. In our study, survival rates in metastatic disease were lower than in the literature. The reason for this may be attributed to the fact that among the patients diagnosed at the metastatic stage, osteosarcoma and rhabdomyosarcoma cases were predominant, which tend to have a worse prognosis compared to other sarcoma types.

Most subtypes of sarcomas have a low tumor mutation burden and are generally considered non-immunogenic tumors [26]. However, this information needs to be re-evaluated, especially with the understanding that the tumor microenvironment can be restructured and is influential in cancer survival. In our study, similar to other studies in the literature, OX40, TIM-3, and LAG-3 were not stained in tumor cells, but staining was observed in the immune microenvironment of the tumor tissue. This suggests that in certain sarcoma subtypes like rhabdomyosarcoma, liposarcoma, and undifferentiated pleomorphic sarcoma, there may be immune escape mechanisms present beyond the tumor microenvironment-associated PD-1/PD-L1 pathway.

In our study, the percentage of PD-L1 in tumor cells is similar to that of some studies in the literature [27,28,29], but it was found to be higher than in others [17,30]. The greatest advantage of our study compared to the existing studies related to PD-L1 is that the number of patients is higher than most, and another valuable aspect is that in addition to PD-L1, OX40, TIM-3, and LAG-3 have also been evaluated. We know from the literature that, especially in non-small-cell lung cancer studies, the expression of PDL-1 and treatment response can vary according to ethnic origin [31,32]. Although ethnic origin and PDL-1 staining have not been evaluated in sarcomas before, we hypothesized that the differences in PDL-1 staining among studies could be attributed to ethnic differences in the patient populations included. Our study observed no significant impact of PD-L1 staining on prognosis, and no relationship was found with overall survival (OS). While there are supportive results in the literature for our study [30,33], a meta-analysis published in 2018 including 10 studies and 911 sarcoma patients, which investigated the relationship between PD-L1 expression and survival, found that PD-L1 expression had a negative impact on survival [5]. Additionally, significant heterogeneity was detected among the studies. Thus, subgroup analyses were conducted. In subgroup analysis, the effect of PD-L1 staining on overall survival (OS) could be observed without heterogeneity in osteosarcoma and soft tissue sarcomas, whereas in the chondrosarcoma group, no effect on OS was observed [5]. In our study, it is thought that the presence of chondrosarcoma patients (8 cases, 7%) may have contributed to the reduction in the impact of PD-L1 on overall survival (OS).

LAG-3 and TIM-3 are receptors that are considered to be highly significant but have not yet been extensively studied in sarcoma. In our study, LAG-3 (36.9%) and TIM-3 (36%) stainings in sarcoma patients were found to be close to the findings of studies in the literature [17,34]. The fact that sarcomas are a tumor type with large tissue volumes, and only a small portion of this can be sampled for staining, along with intra-tumoral heterogeneity, has been considered as a possible reason for the numerical discrepancies. When looking at sarcoma subtypes, similar to the literature, higher levels of LAG-3 and TIM-3 staining were found in patients with undifferentiated pleomorphic sarcoma, rhabdomyosarcoma, and leiomyosarcoma [17].

Our study found no significant relationship between LAG-3 and OS. However, in studies conducted in the literature on this subject, LAG-3 has been identified as a parameter with a negative impact on OS and is considered a poor prognostic factor [34]. Kim et al.’s study published in 2021 evaluated TIM-3 positive CD8 T lymphocytes in the peripheral blood of 86 sarcoma patients and identified its negative prognostic effect. However, to the best of our knowledge, the only study to date that has assessed the TIM-3 receptor in the tumor tissue of sarcoma patients was conducted by Dancsok and colleagues in 2019. In the study, TIM-3 was positive in various subtypes of sarcomas, and co-expression with LAG-3 and PD/PD-L1 was also evaluated [17]. In our study, particularly in rhabdomyosarcoma and UPS, TIM-3 positivity was demonstrated. Additionally, no significant relationship was found between TIM-3 and OS. While this result is significant due to the limited number of studies in the literature, evaluating it in larger patient groups is also important.

In our study, the stage at diagnosis and histological grade were significant independent survival factors, consistent with the literature [22]. Additionally, positive staining of OX-40 was found to have a positive impact on survival in the multivariate analysis. Studies have shown a positive correlation between OX-40 staining in tumor infiltrate and overall survival in non-small-cell lung cancer and colorectal cancer [35,36]. In the literature, our findings are supported by a study conducted in 2010 using a sarcoma-bearing mouse model. In this study, treatment with a novel OX40 ligand-Fc fusion protein was observed to induce tumor regression in the majority of the animals [13]. A study published in 2015 demonstrated that in vitro, OX-40L transgenic Ewing’s sarcoma cells contributed to the enhancement of the immune response and cell death with co-stimulation [14].

T-reg cells express OX40 receptors, which are known to primarily exhibit a diminishing effect on autoimmunity and anti-tumor immune responses. T-reg depletion has been attempted through various receptors (GITR, OX40, CTLA-4, CD25, 4-1BB, etc.) and has been tested in various cancer types to enhance anti-tumor immune responses [37]. However, there are not enough studies focusing on this pathway in sarcomas. In our study, particularly in liposarcoma, rhabdomyosarcoma, and UPS patients, a high level of staining was observed. Therefore, the findings suggest that anti-OX40 treatments could be used in these sarcoma types to enhance anti-tumor immune responses through T-reg depletion.

The most recent and comprehensive study considered to be associated with OX40 in sarcoma patients was conducted on pretreatment pathology specimens of 27 patients who were either unresectable or non-metastatic. This study is also, to the best of our knowledge, the only study in the literature besides our study that evaluates the relationship between OX40 staining in tumor tissue, tumor cells, and/or inflammatory environment and overall survival. In the study, which included patients with UPS, LPS, and myofibroblastic sarcoma, OX40 was detected in Treg cells in tumor tissue both immunohistochemically and transcriptionally [12]. Although OX40 was not found to be prognostic in the conducted analyses, its presence is considered a potential target for new treatments [12]. The limiting factor in studies is the number of patients. To definitively assess its impact on prognosis, studies with a higher number of patients are needed. In our study, although there was no significant contribution of OX40 staining in inflammatory cells in tumor tissue to overall survival (OS) in univariate analysis, patients with staining had a 62-month survival, while those without staining had a 39-month survival. It was also found in the subsequent multivariate analysis evaluation that OX40 positivity had a positive effect on OS. This result emphasizes the importance of OX40 in sarcoma patients and highlights that it could be an important target in this patient group for emerging treatments.

In our study, consistent with the literature, a positive correlation was found between LAG-3, and TIM-3 with PD-L1 staining [17]. While the synergistic effect of LAG-3 and PD-1 on T-cell exhaustion and immune escape has been demonstrated in other tumors, its significance in sarcoma patients is not yet clearly understood [38]. Evaluating PD-L1 and LAG-3 co-expression in sarcoma patients has laid a foundation for future treatment options. In melanoma mouse models resistant to PD-1, the LAG-3 inhibitor Relatlimab is effective against tumor growth. In in vivo experiments, the combination of Relatlimab with the anti-PD-1 antibody was more effective than Relatlimab alone [39]. In sarcoma patients, the first study evaluating a combination of a LAG-3 inhibitor (Relatlimab) with nivolumab is currently ongoing [20]. In a study conducted on head and neck tumors published by Shayan et al. in 2017, it was demonstrated that the expression of TIM-3 increased in tumor-associated lymphocytes of patients who received anti-PD-1 therapy [40]. This has also been considered as an escape mechanism from immune checkpoint inhibitors (ICIs), emphasizing the potential importance of dual blockade therapies. However, there are still not enough studies on the co-expression of TIM-3 and PD/PD-L1 in sarcoma patients. Our study sheds light on future research and treatment approaches in this area. The results of our study highlight that dual or triple immunotherapy blockades, which have been started to be studied in carcinomas, may also be options that should be studied in sarcoma patients in the future.

While many studies conducted on carcinomas have data suggesting that the tumor mutational burden (TMB) positively affects the patient’s response to PD-1/PD-L1 therapy [41,42,43], such evidence is not present in studies conducted on sarcomas [17]. Therefore, there is a need for new biomarkers to determine the patient’s response to immune checkpoint inhibitors (ICI). Sarcomas are a highly heterogeneous group of tumors, and accurate patient selection for treatment will enhance treatment responses. Hence, our study will shed light on whether the simultaneous expression of LAG-3 and TIM-3 with PD-1/PD-L1 expression can act as a biomarker for predicting ICI treatment response in future research.

The most significant limitation of our study is the small sample size, which may be attributed to the tumor’s rarity even in clinical phase studies associated with sarcoma. Another limitation arises from the fact that a diverse range of subtypes within the sarcoma category were evaluated together regarding immunoregulatory receptor expressions due to their low frequency. Differences in T lymphocyte ratios infiltrating the tumor tissue, especially for OX40, TIM-3, and LAG-3, may have played a role in differences between studies. The absence of this in our analysis and evaluations is another limitation. In future studies, placing greater emphasis on subtypes such as UPS, leiomyosarcoma, liposarcoma, and rhabdomyosarcoma, where the expression of immunoregulatory receptors is higher in many studies, will lead to more meaningful results. In our study, we analyzed the relationship between OX40, TIM-3, and LAG-3 with PD-L1. We found a correlation between TIM-3, and LAG-3 with PD-L1 expression, but the correlation between OX40, TIM-3, and LAG-3 was not evaluated in our study. This topic is interesting for future studies.

Until 2019, there were four phase 1-II ICP studies with published results in sarcoma patients. However, as of 2021, this number has increased to fourteen [44,45]. Immunotherapy has been explored in sarcoma patients not only as a monotherapy but also in combination treatments. However, the survival outcomes achieved are still not at the desired level. Therefore, numerous studies are ongoing to determine which patients will benefit from immunotherapy and discover new target pathways in this field [21].

In conclusion, our study has shown that PDL-1, TIM-3, and LAG-3 are expressed at higher rates in certain sarcoma subtypes, and the prognostic impact of OX40 has been identified. Furthermore, a significant positive correlation has been found between PDL-1, TIM-3, and LAG-3 staining. As a result, it has been emphasized that the prolongation of survival times in sarcoma patients in the future can be targeted by using dual or triple blockades with PD-L1’s TİM-3 and LAG-3 inhibitors, and the option of using immunotherapy-targeted treatments together has become a topic of discussion. Future research is needed in sarcoma subgroups to further investigate these findings.

## Figures and Tables

**Table 1:** Characteristics and survival of 111 primary soft tissue and bone sarcoma patients.

Characteristics	Total (n = 111) No. Patients %	
Age		
≤60	84	76
>60	27	24
Gender		
Male	66	59.5
Female	45	40.5
Histology		
Soft tissue sarcoma	68	61.3
Bone sarcoma	43	38.7
Stage		
Local	100	90.1
Metastatic	11	9.9
Tumor site		
Head and neck	13	11.7
Chest	12	10.8
Abdomen	32	28.8
Limb	54	48.6
Tumor size		
≤5 cm	35	31.5
>5 cm	76	68.5
Grade		
Low–intermediate	23	20.7
High	88	79.3
**Median overall survival**	**Months (IQR)**	
Whole group	35 (15–63)	
Metastatic disease group	10 (4–17)	

**Table 2:** Sarcoma types and frequencies.

Types	Frequency	Percent
Leiomyosarcoma	18	16.2
Liposarcoma	12	10.8
Rhabdomyosarcoma	8	7.2
Osteosarcoma	35	31.5
Chondrosarcoma	8	7.2
Synovial sarcoma	7	6.3
Undifferentiated pleomorphic sarcoma	9	8.1
Others	14	12.6
Total	111	100.0

**Table 3:** Percentage of OX40, TIM-3, and Lag-3 staining in lymphocytes and PD-L1 staining in tumor cells.

	OX40 (Positive%)	TİM-3 (Positive%)	LAG-3 (Positive%)	PD-L1 (>1%)
Leiomyosarcoma	22.2%	38.8%	44.4%	16.6%
Liposarcoma	41.6%	41.6%	25%	0%
Rhabdomyosarcoma	50%	75%	50%	37.5%
Osteosarcoma	0%	20%	25.7%	20%
Chondrosarcoma	12.5%	12.5%	37.5%	25%
Synovial sarcoma	14.3%	14.3%	14.3%	14.3%
Undifferentiated pleomorphic sarcoma	33.3%	55.5%	66.6%	33.3%
Total	18%	36%	36.9%	76.6%

**Table 4:** Univariate and multivariate Cox regression models were performed to predict the impact of patient characteristics and staining of OX40, TIM-3, LAG-3, PD-L1 on OS.

Variable	Univariate Analysis			Multivariate Analysis		
	HR	95% CI	*p*-Value	HR	95% CI	*p*-Value
**Age**	0.92	0.50–1.67	0.79			
≤60 years old						
>60 years old						
**Gender**	0.74	0.44–1.26	0.27			
Male						
Female						
**Histology**	1.35	0.80–2.28	0.25			
Soft tissue sarcoma						
Bone sarcoma						
**Stage at diagnosis**	4.7	2.3–9.4	<0.001 *	7.40	3.46–15.8	<0.001 *
Local						
Metastatic						
**Tumor site**	1.15	0.85–1.56	0.36			
Head and neck						
Chest						
Abdomen						
Limb						
**Tumor size**	0.83	0.50–1.41	0.50			
≤5 cm						
>5 cm						
**Grade**	1.81	1.19–2.77	0.001 *	4.3	1.34–3.25	0.001 *
Low–intermediate						
High						
**Pathological staining**	1.01	0.56–1.83	0.95	0.70	0.34–1.46	0.34
**PDL-1**						
0–1%						
<1%						
**OX40**	0.56	0.26–1.18	0.10	0.33	0.14–0.76	0.009 *
Negative						
Positive						
**LAG-3**	0.76	0.44–1.30	0.31	0.71	0.36–1.40	0.32
Negative						
Positive						
**TİM-3**	0.83	0.48–1.42	0.49	1.81	0.94–3.44	0.074
Negative						
Positive						

* Significant.

**Table 5:** The relationship between OX40, TIM-3, and LAG-3 with PD-L1.

	PDL-1 (%)		*p* Value
	0–1%	>1%	
OX40			
Negative n (%)	72 (84.7%)	19 (73.1%)	
Positive n (%)	13 (15.3%)	7 (26.9%)	0.17
TIM-3			
Negative n (%)	61 (71.8%)	10 (38.5%)	
Positive n (%)	24 (28.2%)	16 (61.5%)	0.002 *
LAG-3			
Negative n (%)	63 (74.1%)	7 (26.9%)	
Positive n (%)	22 (25.9%)	19 (73.1%)	<0.001 *

* Significant.

## Data Availability

The datasets generated and analyzed during the current study are not publicly available due to the ‘Patient Rights Regulation’ in our country but are available from the corresponding author upon reasonable request.
